# Six Serum miRNAs Fail to Validate as Myotonic Dystrophy Type 1 Biomarkers

**DOI:** 10.1371/journal.pone.0150501

**Published:** 2016-02-26

**Authors:** Juan M. Fernandez-Costa, Beatriz Llamusi, Ariadna Bargiela, Miren Zulaica, M. Carmen Alvarez-Abril, Manuel Perez-Alonso, Adolfo Lopez de Munain, Arturo Lopez-Castel, Ruben Artero

**Affiliations:** 1 Translational Genomics Group, Incliva Health Research Institute, Valencia, Spain; 2 Department of Genetics and Estructura de Recerca Interdisciplinar en Biotecnologia i Biomedicina (ERI BIOTECMED), Universitat de València, Valencia, Spain; 3 Valentia BioPharma, Scientific Park of the University of Valencia, Valencia, Spain; 4 Neuroscience Area, Biodonostia Institute, Donostia-San Sebastian, Spain; 5 Donostia University Hospital, Donostia-San Sebastian, Spain; SAINT LOUIS UNIVERSITY, UNITED STATES

## Abstract

Myotonic dystrophy type 1 (DM1) is an autosomal dominant genetic disease caused by expansion of a CTG microsatellite in the 3’ untranslated region of the *DMPK* gene. Despite characteristic muscular, cardiac, and neuropsychological symptoms, CTG trinucleotide repeats are unstable both in the somatic and germinal lines, making the age of onset, clinical presentation, and disease severity very variable. A molecular biomarker to stratify patients and to follow disease progression is, thus, an unmet medical need. Looking for a novel biomarker, and given that specific miRNAs have been found to be misregulated in DM1 heart and muscle tissues, we profiled the expression of 175 known serum miRNAs in DM1 samples. The differences detected between patients and controls were less than 2.6 fold for all of them and a selection of six candidate miRNAs, *miR-103*, *miR-107*, *miR-21*, *miR-29a*, *miR-30c*, and *miR-652* all failed to show consistent differences in serum expression in subsequent validation experiments.

## Introduction

Myotonic dystrophy type 1 (DM1) can appear at any time in life and is regarded as the human disease which probably has the most variable clinical presentation, somehow affecting virtually all body systems [1]. Although typically classified as a neuromuscular disease, besides its prominent muscular system defects (including cardiac, smooth, and skeletal muscle cell types), it also compromises cognitive, ocular, digestive, endocrine, respiratory, reproductive, cutaneous, haematopoietic, and immune systems to varying degrees [2]. Characteristic muscular symptoms include cardiac problems such as malignant arrhythmias and conduction defects, and involvement of facial (ptosis), bulbar (dysarthria, dysphagia), limb (steppage, gait troubles), and smooth (constipation) muscle with associated muscular atrophy and myotonia [1, 3, 4]. Patients also suffer from iridescent cataracts and insulin resistance with metabolic syndrome. Genetically, it is an autosomal dominant disease caused by unstable expansion of the CTG microsatellite in the 3’ untranslated region of the *dystrophia myotonica-protein kinase* (*DMPK*) gene and is a rare disease that afflicts one in 8000 people worldwide. Unaffected individuals carry between 5 and 37 CTG repeats whereas DM1 patients carry between 50 and thousands of CTG triplets [5]. Importantly, CTG trinucleotide expansions are unstable both in the somatic and germinal lines, likely contributing to the heterogeneity in clinical symptoms and age of onset, which inversely correlates with the size of the triplet expansion. A further increase in the size of the CTG microsatellite occurs in most intergenerational transmissions of the expanded allele, which correlates with genetic anticipation [6]. Despite the correlation between the size of the CTG expansions in blood cells with disease severity and age of onset, its predictive power is poor and it is not a good parameter for characterising the disease load. Forthcoming therapeutic trials urgently need good biomarkers to evaluate the therapeutic response to treatments. Alternative splicing changes in skeletal muscle have been described as potential biomarkers of disease severity and therapeutic response, but they involve invasive techniques [7] and it would be difficult to routinely measure them in other sites (such as cardiac or cerebral tissues) which are strongly involved in DM1 pathophysiology.

Expanded RNA transcripts containing CUG repeats are retained in the cell nucleus as insoluble RNA aggregates known as ribonuclear foci [8]. These foci are able to sequester different RNA binding proteins that are prevented from performing their normal functions. The alternative splicing regulators Muscleblind-like1 (MBNL1) is among the recruited proteins, which result in its functional depletion [9]. CUGBP, Elav-like family member 1 (CELF1), a splicing factor antagonist of MBNL1 [10], is not sequestered in ribonuclear foci but becomes abnormally activated due to hyperphosphorylation [11]. As a consequence, several alternative splicing events are misregulated in DM1 and in some cases these splicing defects contribute to DM1 symptoms such as myotonia, insulin resistance, or muscle weakness [7, 12, 13, 14]. The molecular mechanism leading to DM1 pathogenesis is complex and, in addition to splicing defects, also includes mispolyadenylation of pre-mRNA, a process that is also regulated by MBNL proteins [15], repeat-associated non-ATG translation (RAN translation) [16], bidirectional transcription [17], defects in transcription and translation [18, 19], epigenetic changes [20], and the silencing of cardiac and muscle transcripts by changes in miRNA expression levels [21, 22, 23, 24]. miRNAs are endogenous non-coding RNAs, approximately 21 nucleotides long, that function as post-transcriptional gene expression regulators by targeting the 3’ untranslated region of their complementary target mRNA. miRNAs regulate RNA stability and translation rates via degradation or inhibition of protein translation, respectively (reviewed in [25]). Over 2000 miRNAs have been identified in the human genome [26] and have been implicated in numerous biological processes including development, proliferation, differentiation, and stress responses (reviewed in [27]). Because miRNAs can be readily detected in body fluids, and particularly in blood components [28], differences in serum miRNAs have been proposed as potential non-invasive biomarkers of disease progression for several conditions such as cancer, Alzheimer´s disease, hepatitis B infection, retinopathies, gestational diabetes mellitus, or Duchenne muscular dystrophy [29, 30, 31, 32, 33, 34].

Because several miRNAs have been detected to be altered in DM1 cardiac and muscle tissues [21, 22, 23, 24, 35], and there are numerous drugs that work in DM1 animal models pending accurate pharmacological development and clinical testing in humans [36, 37, 38, 39], we explored the possibility that misexpression of specific serum miRNAs could be identified as non-invasive DM1 biomarkers. To this end, we profiled 175 miRNAs in the peripheral blood serum of DM1-affected individuals and healthy controls by real time qPCR. Even though none of them showed expression differences greater than 2.6 fold, the six miRNAs with the highest fold-change score (*miR-103*, *miR-107*, *miR-21*, *miR-29a*, *miR-30c*, and *miR-652*) were further investigated but no significant differences between the control and DM1 conditions were found for any of them.

## Materials and Methods

### Sample collection and serum isolation

This study was approved by the Ethics Committee at the University of Valencia. All blood samples were taken after specific written informed consent to participate in the present study. All individuals were Subjects with DM1 were ambulatory adults with proven CTG expansions. Peripheral blood samples were obtained by venous punctures with a fine bore needle (21 G ¾”) of 26 DM1 and 22 healthy individuals (Tables 1 and 2) and placed in serum collection tubes (BD VACUTAINER SST II ADVANCE). After 10 min centrifugation at 1200 g at room temperature, the serum was aliquoted and kept at -80°C until use. For CTG repeat size determination, genomic DNA was isolated from peripheral blood leucocytes [40] and was processed for Southern blotting with a ^32^P-labelled cDNA25 probe (S1 Fig) or, alternatively, the CTG-repeat region was amplified by PCR using DM101 and DM102 as primers [41, 42, 43, 44].

**Table 1:** Information about the samples used in the miRNA profiling.

sample	sex	age	(CTG)n	Sample	sex	age	(CTG)n
**P_1**	male	48	667	**C_11**	male	40	-
**P_2**	male	41	500	**C_12**	male	53	-
**P_3**	male	53	800	**C_13**	male	44	-
**P_4**	male	50	333	**C_14**	male	54	-
**P_5**	male	54	333	**C_15**	male	45	-
**P_6**	male	56	333	**C_16**	male	42	-
**P_7**	male	48	333	**C_17**	male	43	-
**P_8**	male	57	1333	**C_18**	male	53	-
**P_9**	male	56	1000	**C_19**	male	41	-
**P_10**	male	50	333	**C_20**	male	46	-

**Table 2:** Information about the samples used in qPCR.

sample	sex	age	(CTG)n	sample	sex	age	(CTG)n
**P_1**	male	48	667	**C_11**	male	40	-
**P_2**	male	41	500	**C_12**	male	53	-
**P_4**	male	50	333	**C_13**	male	44	-
**P_5**	male	54	333	**C_14**	male	54	-
**P_6**	male	56	333	**C_15**	male	45	-
**P_7**	male	48	333	**C_16**	male	42	-
**P_8**	male	57	1333	**C_17**	male	43	-
**P_9**	male	56	1000	**C_19**	male	41	-
**P_10**	male	50	333	**C_20**	male	46	-
**21**	male	56	70	**50**	male	45	--
**22**	female	61	333	**51**	male	58	--
**23**	female	44	1000	**52**	male	49	--
**24**	female	53	730	**53**	male	52	--
**25**	female	41	500	**54**	female	59	--
**26**	female	42	833	**55**	female	59	--
**28**	female	46	667	**57**	female	57	--
**30**	female	45	667	**58**	female	65	--
**32**	female	48	1333	**60**	female	61	--
**33**	male	26	1000	**61**	female	55	--
**34**	male	38	1333	**62**	female	51	--
**35**	male	37	1000	**64**	male	34	--
**36**	male	39	1333	**65**	female	25	--
**37**	male	31	333				
**38**	male	36	400				
**39**	male	30	333				
**40**	male	37	333				

### RNA extraction and cDNA synthesis

We assayed for the presence of oxyhaemoglobin in the serum samples because haemolysis has been described to affect the levels of certain miRNAs [45]. The absorbance at 414 nm was determined spectrophotometrically and samples with an absorbance higher than 0.2 were discarded, as this is the cutoff at which samples have previously been considered to be haemolysed [45]. Independent total RNA extraction was performed for each serum sample using the miRNeasy Mini kit (Qiagen). Briefly, 500 μL of serum was thawed on ice, centrifuged for 5 min at 3000 g at 4°C and 200 μL of the supernatant serum was taken and mixed with 750 μL QIAzol containing 1.25 mg/mL bacteriophage MS2 RNA as a carrier. The extraction was performed according to the manufacturer’s instructions, except that the final wash (with RPE buffer) was performed three times instead of once. Total RNA was eluted with 50 μL water, and cDNA synthesis was performed with 4 μL of total RNA using the Universal cDNA synthesis II kit (Exiqon).

### MicroRNA expression profiling and validation

The miRCURY LNA™ Universal RT microRNA PCR assay and the Serum/Plasma Focus microRNA PCR Panel (Exiqon) was used for miRNA expression profiling. These panels contain primers for the detection of the 175 most-expressed miRNAs in human serum (S1 Table). Each 384-well plate contained 2 complete panels of primers and 2 negative controls; real-time PCR was performed according to the manufacturer’s instructions, and cDNAs from a DM1 patient and a control sample were amplified in parallel in each plate. Expression values were calculated using the 2^-∆∆Ct^ method [46] using the mean Ct of miRNAs detected (Ct < 34) for normalisation (S1 Table). During the validation step, the analysis of expression of these miRNAs used real-time PCRs with specific miRCURY LNA microRNA PCR primers (Exiqon). The GeNorm and Normfinder algorithms were used to find optimal reference genes to normalise the expression of the miRNAs being validated [47, 48]. Expression level determinations were performed using an Applied Biosystems 7900HT Fast Real-Time PCR System.

### Statistical analysis

A logarithmic transformation (log2) was used to normalise the expression data in the profiling experiment. Expression differences were analysed using the Student t-test and different methods for multiple-testing correction were applied, including Bonferroni, Benjamini-Hochberg (False Discovery Rate), Westfall-Young, and Benjamini-Yekutieli corrections. Cluster software was used for hierarchical clustering analysis of genes and samples. Euclidean distances and the average linkage method were selected, using the normalised expression values of each miRNA to represent clusters.

## Results

### MicroRNA expression profiling in human myotonic dystrophy type 1 serum

**Fig 1:**
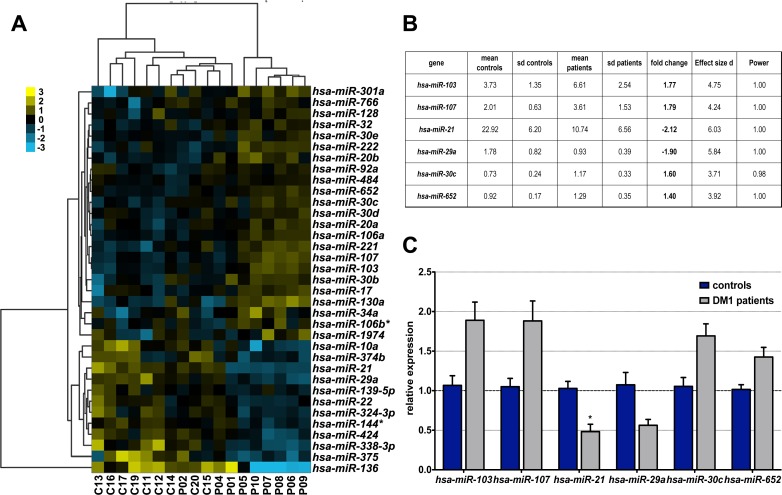
Profiling of miRNA expression levels in myotonic dystrophy type 1 patients and controls. (A) Heat map graphical representation and clustering analysis of miRNA expression from 9 DM1 patients (P01-P10, excluding P03) and 9 healthy controls (C11-C20 excluding C18). Blue and yellow indicate statistically significant down- and upregulated miRNAs compared to controls, respectively (t-test α  =  0.05). Data is presented as a dendrogram, with the closest branches of the tree showing samples with less dissimilar expression patterns. (B) Statistical analysis of the miRNA profiling carried out with the G*Power tool. These miRNAs have the highest fold-change and Power∼1 statistics in the sample pool. (C) Graphical representation of the expression levels of the miRNAs selected via G*Power analysis. Only *miR-21* showed a statistically-significant difference when Bonferroni correction was applied. Graph bars represent average fold changes and their standard errors. *P* > 0.05.

A total of 175 miRNA expression levels were obtained from each peripheral blood serum total-RNA sample using commercial microRNA PCR panels (Exiqon; S1 Table). miRNA profiling was initially carried out with peripheral blood from 10 male DM1-affected individuals (aged 51.3 ± 1.6; P01-P10), expressing between 333 and 1333 CTG repeats (in blood samples), and 10 sex and age-matched controls (aged 46.1 ± 1.7; C11-C20) that did not display any neuromuscular disorders (Table 1). The absorbance at 414 nm was measured in all the samples to discard the possibility of haemolysis, which can occur during blood collection and has a potentially substantial impact on serum miRNA content [49] (S2 Table). Because two samples, P3 and C18, generated a positive result for this parameter (absorbance > 0.2) they were discarded during data analysis (S3 Table). Expression data from each sample was initially normalised to the mean values of all 175 miRNAs. Statistical analysis of the results (Student t-test) showed 35 miRNAs with a *P*-value lower than 0.05, of which 24 miRNAs were up- and 11 were downregulated when compared to controls (Fig 1A, S3 Table). However, only miR-21 was significantly downregulated in DM1 according to three different statistical corrections (Bonferroni, Benjamini-Hochberg (False Discovery Rate) and Westfall-Young, S3 Table). It is worth mentioning that all the differences in expression levels detected between controls and DM1 patients were relatively low (below 2.6-fold) compared to other described biomarkers [50, 51, 52]. Given the controversy regarding the most appropriate way to normalise data when determining miRNA expression values, in addition to mean normalisation, we also normalised the data to specific miRNA expression levels [53]. For that purpose we used two different algorithms, NormFinder and geNorm [47, 48], to identify the most stable miRNAs from our study cohort (S4 Table). Therefore we normalised the *miR-15a*, *miR-23a*, *miR-28-3p*, and *miR-484* expression levels to the mean of *miR-15a*, *miR-23a*, and *miR-484* and the mean of *miR-15a* and *miR-28-3p* (S3 Table). In most cases, *miR-21* was the only miRNA with significantly different expression between the controls and patients.

We carried out additional statistical analyses using G*Power software to select additional candidate miRNAs to validate by qPCR. We chose miRNAs with the highest fold-change and with a Power value ∼1, which included *miR-21*. Considering these parameters, we selected the six most promising miRNAs for validation: *miR-103*, *miR-107*, *miR-21*, *miR-29a*, *miR-30c*, and *miR-652* (Fig 1B and 1C).

### Expression quantification of six candidate miRNAs in serum

**Fig 2:**
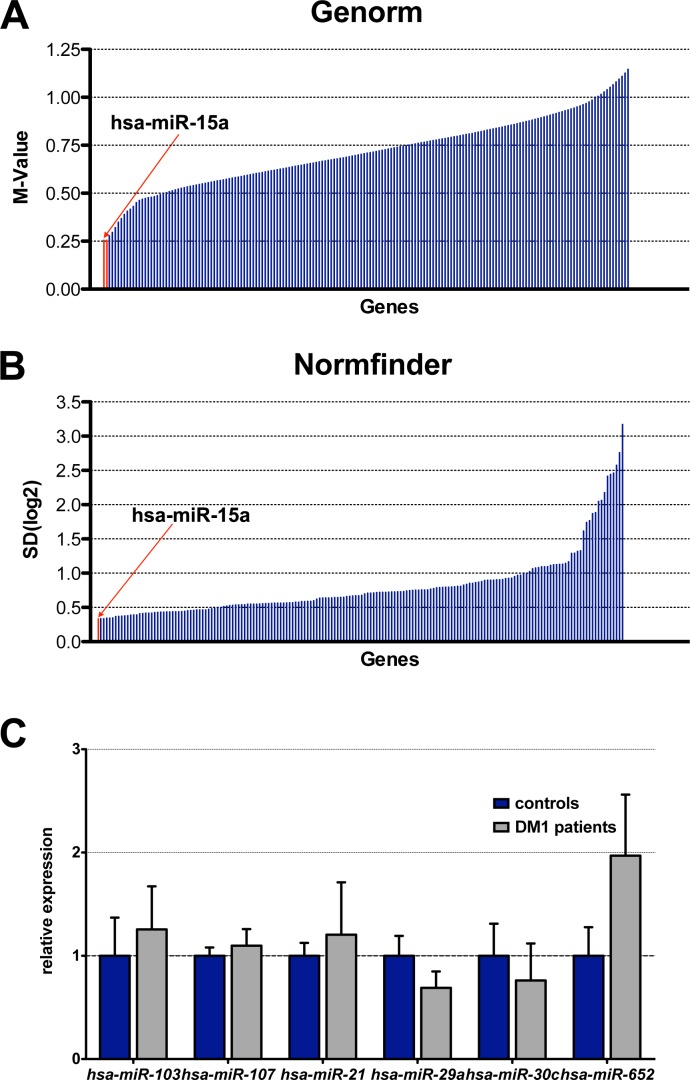
Validation by q-PCR did not reveal differences in miRNA expression levels between controls and myotonic dystrophy type 1 patients. (A, B) Graphical representation of the results generated by two algorithms, geNorm and NormFinder, to identify the optimal normalisation miRNA from among all of the candidates (S4 Table). (C) Analysis of the relative expression levels of *miR-103*, *miR-107*, *miR-21*, *miR-29a*, *miR-30c*, and *miR-652* by quantitative PCR on the serum samples of nine DM1 patients and nine healthy individuals. All data were normalised to *miR-15* expression levels but no significant differences were observed between either group. Graph bars represent average fold-changes of miRNA expression on a logarithmic scale, calculated using the 2^-∆∆Ct^ method, as well as their confidence intervals. Graph bars represent average fold changes of miRNA expression, calculated using the 2^-∆∆Ct^ method, along with their standard error.

We experimentally determined individual expression levels of *miR-103*, *miR-107*, *miR-21*, *miR-29a*, *miR-30c*, and *miR-652* in the same nine DM1 and nine control serum samples used during the initial profiling. The data were normalised to *miR-15a*, the most and second-most stable miRNA from all of the samples, according to geNorm and NormFinder, respectively (Fig 2A and 2B; S4 Table). However, we did not detect statistically-significant differences between DM1 and control samples, not even for *miR-21*, the only miRNA that was positive after the profiling (Fig 2C).

**Fig 3:**
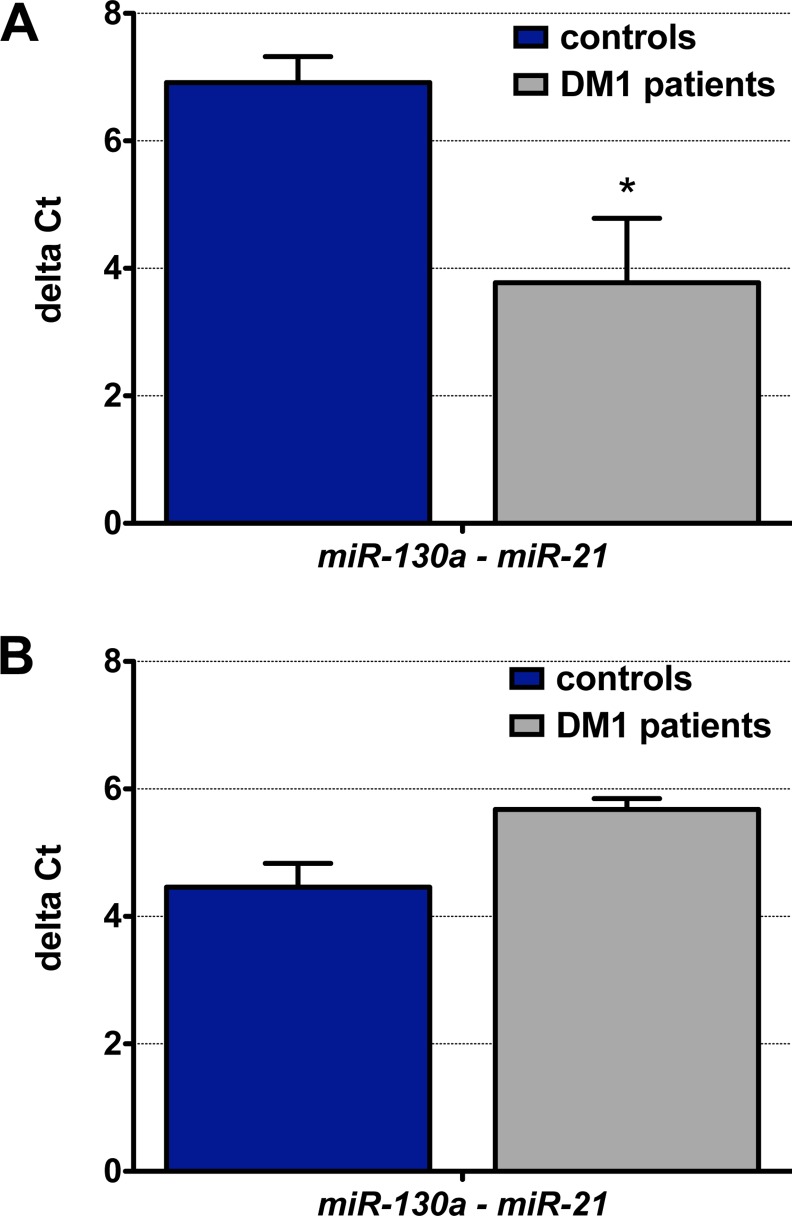
The ratio of *miR-130a* and *miR-21* failed as a myotonic dystrophy type 1 biomarker. (A) The ratio of *miR-130a* and *miR-21* according to expression levels obtained from the profiling performed with serum samples from nine DM1 patients and nine healthy controls. (B) The same ratio was calculated after measuring *miR-130a* and *miR-21* expression levels by quantitative PCR on serum samples from 21 DM1 and 17 control individuals. No statistically-significant differences were observed. Graph bars represent the average ∆Cts (*miR-130a*--*miR-2*1) and their standard errors.

Considering that from among all of our results, data supporting *miR-21* misexpression was the strongest, we decided to carry out further analyses on it. To prevent potential false-negative results because of the miRNA selected as a normaliser, we normalised *miR-21* to the expression of the miRNA with the strongest alteration in the opposite direction because this ratio would be independent of any endogenous control. Taking into account data from the profiling (S1 Table), *miR-21* expression was downregulated 2.4 times in DM1 samples while *miR-130a* was upregulated, with a 2.5-fold change (Fig 3A). Next, we used serum samples from 21 DM1 male and female individuals and 17 counterpart controls to quantify expression levels of *miR-21* and *miR-130a*. We confirmed the absence of haemolysis in all the samples by measuring absorbance at 414 nm (S2 Table). However, again, we were unable to detect any significant difference in the *miR-21* to *miR-130a* ratio between controls and DM1 samples (Fig 3B).

## Discussion

The only method available for monitoring the progression of DM1 is clinical assessment provided by semiquantitative scales, which correlates poorly with underlying biological defects [54]. A more targeted strategy which characterises muscle involvement is the transcriptomic analysis of muscle biopsies via invasive techniques. These analyses have led to the recent proposal that suggests that alternative splicing events in skeletal muscle can serve as valid biomarkers for quantifying the severity of DM1 and its likely response to therapy [14]. Nevertheless, the different patterns of muscle involvement in DM1, and the invasive nature of the approach, inherently limits this proposal as a good measurement of outcome. An alternative method for neuromuscular diseases is to use blood miRNAs as biomarkers. Cacchiarelli et al. described three miRNAs that correlated with disease severity in Duchenne muscular dystrophy where, as a consequence of muscle-fibre damage, muscle miRNAs are released into the bloodstream [34]. However, cell membranes remain undamaged in DM1 muscle fibres [55] and, consequently, levels of myomiRs in blood in these patients are not expected to be as dramatically increased as a result of the disease as in Duchenne muscular dystrophy.

In the present work we profiled 175 miRNAs in serum samples but we did not observe differences greater than 2.6-fold between DM1 patients and healthy individuals. Owing to the novelty in the use of miRNAs as biomarkers, there is a lack of consensus regarding different technical aspects such as sample quality [56]. However, it was demonstrated that haemolysis can occur during blood collection and this can have a substantial effect on miRNA content in plasma or serum [49]. This fact shows the relevance of good sample quality control for the results obtained when searching for miRNA biomarkers. Initially, we performed the profiling with ten control and ten DM1 patient samples, however one sample from each group was removed because of unacceptable haemolysis levels. After the profiling we identified 35 altered candidate-miRNAs. However, after applying Bonferroni correction only one, *miR-21*, was statistically different.

It was recently published that four muscle-specific miRNAs, *miR-1*, *miR-133a*, *miR-133b*, and *miR-206*, were altered in serum from DM1 patients [57]. Of these, *miR-1*, *miR-133a*, and *miR-133b* were included in our profiling panel, however, we did not detect differences in their expression levels. There are two aspects that should be considered: Firstly, that in this aforementioned work the data were normalised to *miR-16* expression, however other work has shown that levels of this miRNA vary as a function of haemolysis levels because it is one of the most abundant miRNAs in red blood cells [45, 49, 58], and thus *miR-16* levels may be unacceptably influenced by haemolysis. Moreover, it was recently observed that *miR-16* is sequestered by long CUG repeats [59] and consequently the amount of free *miR-16* in the bloodstream may not be equal in healthy and DM1-affected individuals. Secondly, different methods may identify different sets of altered miRNAs. Indeed, a recent review showed that plasma and serum miRNAs described as breast cancer biomarkers in different publications in the literature do not overlap with each other [60], and although the exact reasons remain unclear, methodological differences in experimental procedures may be one major cause [61].

Another group, using a similar approach to ours, identified nine miRNAs that were differentially expressed between healthy controls and DM1 individuals [62]. Of those, seven were included in our profiling panel, however, none of them were positive. Of note, Perfetti et al. used plasma samples in their research, whereas we used serum; this is worth mentioning because differences in miRNA and RNA levels in serum vs. plasma have been reported [28]. The authors suggested that miRNAs are released from blood cells into serum during the coagulation process, although they did not identify the reason for this. Therefore, results regarding the biomarkers identified using serum vs. plasma are not comparable. It is also noteworthy that we used different measurement platforms to those employed by Perfetti et al.: the Taqman and Exiqon miRNA qPCR panels, respectively. In this regard, Wang et al. demonstrated that the consistency between results obtained using both platforms is low [28], finding that from 358 miRNAs, only ∼19% were detected by both platforms, and that Taqman measurements were 6.7 Ct-values higher than those from Exiqon [28].

After miRNA profiling we identified only one miRNA that was differentially expressed: *miR-21*. We tried to validate five additional miRNAs by qPCR, although the differences between controls and DM1 were not statistically significant, and we did not observe differential expression in any case. qPCR data were normalised to *miR-15a* because two different algorithms identified it as the most stable miRNA from among all of the samples. In addition, *miR-21* expression was assessed as a ratio to *miR-130a*, however, neither of these analyses revealed significant differences. We used serum samples from males in the profiling, and samples from both genders during the validation where a higher number of samples was needed. However, it is unlikely this had any significant effect on our results because the qPCR results for each gender were similar to the results obtained from the combined sample analysis.

In summary, we conclude that, under our reported conditions, the miRNAs *miR-103*, *miR-107*, *miR-21*, *miR-29a*, *miR-30c*, and *miR-652* are not useful serum biomarkers for DM1. Although the successful use of miRNAs from body fluids as disease-severity and progression biomarkers in other studies represents an encouraging advance, several technical aspects must first be standardised because methodological differences in the experimental procedures seem to be the main reason that data from different studies do not coincide [61].

## Supporting Information

S1 Fig Representative example of Southern blot used for CTG repeat size determination. Arrows indicate wild type alleles. Mutant alleles in patients 1,3, 4 and 5 present more than 1000 CTG repeats while in patient 2 the mutant allele has a a repeat size of ~600 repeats.
(TIF)

S1 Table Cts values for 175 miRNAs in DM1 and controls serum. (XLS)

S2 Table Absorbance at 414 nm for sera used in the assay. (XLS)

S3 Table Statitical analyses. (XLS)

S4 Table Genorm and Normfinder algorithms output for most stable miRNAs in the study. (XLS)

## References

[1] Harper P. Myotonic dystrophy London: Saunders; 2001.

[2] Gagnon C, Noreau L, Moxley RT, Laberge L, Jean S, Richer L, et al (2007) Towards an integrative approach to the management of myotonic dystrophy type 1. J Neurol Neurosurg Psychiatry 78: 800–6. 17449544 10.1136/jnnp.2006.107185 PMC2117723

[3] Petri H, Vissing J, Witting N, Bundgaard H, Kober L. (2012) Cardiac manifestations of myotonic dystrophy type 1. Int J Cardiol 160: 82–8. 10.1016/j.ijcard.2011.08.037 21917328

[4] Ercolin B, Sassi FC, Mangilli LD, Mendonca LI, Limongi SC, de Andrade CR. (2013) Oral motor movements and swallowing in patients with myotonic dystrophy type 1. Dysphagia 28: 446–54. 10.1007/s00455-013-9458-9 23460343

[5] Ashizawa T, Sarkar PS. (2011) Myotonic dystrophy types 1 and 2. Handb Clin Neurol 101: 193–237. 10.1016/B978-0-08-045031-5.00015-3 21496635

[6] Lopez de Munain A, Cobo A, Marti Masso JF, Baiget M. (1994) [DNA instability and neurological diseases: a new model for genetic disease]. Neurologia 9: 342–51. 7803051

[7] Mankodi A, Takahashi MP, Jiang H, Beck CL, Bowers WJ, Moxley RT, et al (2002) Expanded CUG repeats trigger aberrant splicing of ClC-1 chloride channel pre-mRNA and hyperexcitability of skeletal muscle in myotonic dystrophy. Mol Cell 10: 35–44. 12150905 10.1016/s1097-2765(02)00563-4

[8] Davis BM, McCurrach ME, Taneja KL, Singer RH, Housman DE. (1997) Expansion of a CUG trinucleotide repeat in the 3' untranslated region of myotonic dystrophy protein kinase transcripts results in nuclear retention of transcripts. Proc Natl Acad Sci U S A 94: 7388–93. 9207101 10.1073/pnas.94.14.7388 PMC23831

[9] Fardaei M, Rogers MT, Thorpe HM, Larkin K, Hamshere MG, Harper PS, et al (2002) Three proteins, MBNL, MBLL and MBXL, co-localize in vivo with nuclear foci of expanded-repeat transcripts in DM1 and DM2 cells. Hum Mol Genet 11: 805–14. 11929853 10.1093/hmg/11.7.805

[10] Wang ET, Ward AJ, Cherone JM, Giudice J, Wang TT, Treacy DJ, et al (2015) Antagonistic regulation of mRNA expression and splicing by CELF and MBNL proteins. Genome Res 25: 858–71. 10.1101/gr.184390.114 25883322 PMC4448682

[11] Kuyumcu-Martinez NM, Wang GS, Cooper TA. (2007) Increased steady-state levels of CUGBP1 in myotonic dystrophy 1 are due to PKC-mediated hyperphosphorylation. Mol Cell 28: 68–78. 17936705 10.1016/j.molcel.2007.07.027 PMC2083558

[12] Du H, Cline MS, Osborne RJ, Tuttle DL, Clark TA, Donohue JP, et al (2010) Aberrant alternative splicing and extracellular matrix gene expression in mouse models of myotonic dystrophy. Nat Struct Mol Biol 17: 187–93. 10.1038/nsmb.1720 20098426 PMC2852634

[13] Suenaga K, Lee KY, Nakamori M, Tatsumi Y, Takahashi MP, Fujimura H, et al (2012) Muscleblind-like 1 knockout mice reveal novel splicing defects in the myotonic dystrophy brain. PLoS One 7: e33218 10.1371/journal.pone.0033218 22427994 PMC3302840

[14] Nakamori M, Sobczak K, Puwanant A, Welle S, Eichinger K, Pandya S, et al (2013) Splicing biomarkers of disease severity in myotonic dystrophy. Ann Neurol 74: 862–72. 10.1002/ana.23992 23929620 PMC4099006

[15] Batra R, Charizanis K, Manchanda M, Mohan A, Li M, Finn DJ, et al (2014) Loss of MBNL leads to disruption of developmentally regulated alternative polyadenylation in RNA-mediated disease. Mol Cell 56: 311–22. 10.1016/j.molcel.2014.08.027 25263597 PMC4224598

[16] Cleary JD, Ranum LP. (2013) Repeat-associated non-ATG (RAN) translation in neurological disease. Hum Mol Genet 22: R45–51. 10.1093/hmg/ddt371 23918658 PMC3782068

[17] Nakamori M, Pearson CE, Thornton CA. (2011) Bidirectional transcription stimulates expansion and contraction of expanded (CTG)*(CAG) repeats. Hum Mol Genet 20: 580–8. 10.1093/hmg/ddq501 21088112 PMC3016912

[18] Ebralidze A, Wang Y, Petkova V, Ebralidse K, Junghans RP. (2004) RNA leaching of transcription factors disrupts transcription in myotonic dystrophy. Science 303: 383–7. 14657503 10.1126/science.1088679

[19] Kim YK, Mandal M, Yadava RS, Paillard L, Mahadevan MS. (2014) Evaluating the effects of CELF1 deficiency in a mouse model of RNA toxicity. Hum Mol Genet 23: 293–302. 10.1093/hmg/ddt419 24001600 PMC3924053

[20] Lopez Castel A, Nakamori M, Tome S, Chitayat D, Gourdon G, Thornton CA, et al (2011) Expanded CTG repeat demarcates a boundary for abnormal CpG methylation in myotonic dystrophy patient tissues. Hum Mol Genet 20: 1–15. 10.1093/hmg/ddq427 21044947 PMC3000673

[21] Perbellini R, Greco S, Sarra-Ferraris G, Cardani R, Capogrossi MC, Meola G, et al (2011) Dysregulation and cellular mislocalization of specific miRNAs in myotonic dystrophy type 1. Neuromuscul Disord 21: 81–8. 10.1016/j.nmd.2010.11.012 21169019

[22] Fernandez-Costa JM, Garcia-Lopez A, Zuniga S, Fernandez-Pedrosa V, Felipo-Benavent A, Mata M, et al (2013) Expanded CTG repeats trigger miRNA alterations in Drosophila that are conserved in myotonic dystrophy type 1 patients. Hum Mol Genet 22: 704–16. 10.1093/hmg/dds478 23139243

[23] Kalsotra A, Singh RK, Gurha P, Ward AJ, Creighton CJ, Cooper TA. (2014) The Mef2 transcription network is disrupted in myotonic dystrophy heart tissue, dramatically altering miRNA and mRNA expression. Cell Rep 6: 336–45. 10.1016/j.celrep.2013.12.025 24412363 PMC3927417

[24] Rau F, Freyermuth F, Fugier C, Villemin JP, Fischer MC, Jost B, et al (2011) Misregulation of miR-1 processing is associated with heart defects in myotonic dystrophy. Nat Struct Mol Biol 18: 840–5. 10.1038/nsmb.2067 21685920

[25] Valinezhad Orang A, Safaralizadeh R, Kazemzadeh-Bavili M. (2014) Mechanisms of miRNA-Mediated Gene Regulation from Common Downregulation to mRNA-Specific Upregulation. Int J Genomics 2014: 970607 10.1155/2014/970607 25180174 PMC4142390

[26] Kozomara A, Griffiths-Jones S. (2014) miRBase: annotating high confidence microRNAs using deep sequencing data. Nucleic Acids Res 42: D68–73. 10.1093/nar/gkt1181 24275495 PMC3965103

[27] Iwakawa HO, Tomari Y. (2015) The Functions of MicroRNAs: mRNA Decay and Translational Repression. Trends Cell Biol. 10.1016/j.tcb.2015.07.011 26437588

[28] Wang K, Yuan Y, Cho JH, McClarty S, Baxter D, Galas DJ. (2012) Comparing the MicroRNA spectrum between serum and plasma. PLoS One 7: e41561 10.1371/journal.pone.0041561 22859996 PMC3409228

[29] Hennessey PT, Sanford T, Choudhary A, Mydlarz WW, Brown D, Adai AT, et al (2012) Serum microRNA biomarkers for detection of non-small cell lung cancer. PLoS One 7: e32307 10.1371/journal.pone.0032307 22389695 PMC3289652

[30] Geekiyanage H, Jicha GA, Nelson PT, Chan C. (2012) Blood serum miRNA: non-invasive biomarkers for Alzheimer's disease. Exp Neurol 235: 491–6. 10.1016/j.expneurol.2011.11.026 22155483 PMC3361462

[31] Chen Y, Li L, Zhou Z, Wang N, Zhang CY, Zen K. (2012) A pilot study of serum microRNA signatures as a novel biomarker for occult hepatitis B virus infection. Med Microbiol Immunol 201: 389–95. 10.1007/s00430-011-0223-0 22392036

[32] Qing S, Yuan S, Yun C, Hui H, Mao P, Wen F, et al (2014) Serum miRNA biomarkers serve as a fingerprint for proliferative diabetic retinopathy. Cell Physiol Biochem 34: 1733–40. 10.1159/000366374 25427542

[33] Zhao C, Dong J, Jiang T, Shi Z, Yu B, Zhu Y, et al (2011) Early second-trimester serum miRNA profiling predicts gestational diabetes mellitus. PLoS One 6: e23925 10.1371/journal.pone.0023925 21887347 PMC3161072

[34] Cacchiarelli D, Legnini I, Martone J, Cazzella V, D'Amico A, Bertini E, et al (2011) miRNAs as serum biomarkers for Duchenne muscular dystrophy. EMBO Mol Med 3: 258–65. 10.1002/emmm.201100133 21425469 PMC3112257

[35] Gambardella S, Rinaldi F, Lepore SM, Viola A, Loro E, Angelini C, et al (2010) Overexpression of microRNA-206 in the skeletal muscle from myotonic dystrophy type 1 patients. J Transl Med 8: 48 10.1186/1479-5876-8-48 20487562 PMC2880982

[36] Garcia-Lopez A, Llamusi B, Orzaez M, Perez-Paya E, Artero RD. (2011) In vivo discovery of a peptide that prevents CUG-RNA hairpin formation and reverses RNA toxicity in myotonic dystrophy models. Proc Natl Acad Sci U S A 108: 11866–71. 10.1073/pnas.1018213108 21730182 PMC3141925

[37] Pandey SK, Wheeler TM, Justice SL, Kim A, Younis HS, Gattis D, et al (2015) Identification and Characterization of Modified Antisense Oligonucleotides Targeting DMPK in Mice and Nonhuman Primates for the Treatment of Myotonic Dystrophy Type 1. J Pharmacol Exp Ther 355: 310–21. 10.1124/jpet.115.226969 PMC4613955 26330536

[38] Parkesh R, Childs-Disney JL, Nakamori M, Kumar A, Wang E, Wang T, et al (2012) Design of a bioactive small molecule that targets the myotonic dystrophy type 1 RNA via an RNA motif-ligand database and chemical similarity searching. J Am Chem Soc 134: 4731–42. 10.1021/ja210088v 22300544 PMC3306011

[39] Wheeler TM, Leger AJ, Pandey SK, MacLeod AR, Nakamori M, Cheng SH, et al (2012) Targeting nuclear RNA for in vivo correction of myotonic dystrophy. Nature 488: 111–5. 10.1038/nature11362 22859208 PMC4221572

[40] Miller SA, Dykes DD, Polesky HF. (1988) A simple salting out procedure for extracting DNA from human nucleated cells. Nucleic Acids Res 16: 1215 3344216 10.1093/nar/16.3.1215 PMC334765

[41] Aslanidis C, Jansen G, Amemiya C, Shutler G, Mahadevan M, Tsilfidis C, et al (1992) Cloning of the essential myotonic dystrophy region and mapping of the putative defect. Nature 355: 548–51. 1346925 10.1038/355548a0

[42] Brook JD, McCurrach ME, Harley HG, Buckler AJ, Church D, Aburatani H, et al (1992) Molecular basis of myotonic dystrophy: expansion of a trinucleotide (CTG) repeat at the 3' end of a transcript encoding a protein kinase family member. Cell 69: 385. 10.1016/0092-8674(92)90418-c 1568252

[43] Buxton J, Shelbourne P, Davies J, Jones C, Van Tongeren T, Aslanidis C, et al (1992) Detection of an unstable fragment of DNA specific to individuals with myotonic dystrophy. Nature 355: 547–8. 1346924 10.1038/355547a0

[44] Erginel-Unaltuna N, Akbas F. (2004) Improved method for molecular diagnosis of myotonic dystrophy type 1 (DM1). J Clin Lab Anal 18: 50–4. 14730559 10.1002/jcla.20004 PMC6807958

[45] Kirschner MB, Kao SC, Edelman JJ, Armstrong NJ, Vallely MP, van Zandwijk N, et al (2011) Haemolysis during sample preparation alters microRNA content of plasma. PLoS One 6: e24145 10.1371/journal.pone.0024145 21909417 PMC3164711

[46] Pfaffl MW, Horgan GW, Dempfle L. (2002) Relative expression software tool (REST) for group-wise comparison and statistical analysis of relative expression results in real-time PCR. Nucleic Acids Res 30: e36 11972351 10.1093/nar/30.9.e36 PMC113859

[47] Vandesompele J, De Preter K, Pattyn F, Poppe B, Van Roy N, De Paepe A, et al (2002) Accurate normalization of real-time quantitative RT-PCR data by geometric averaging of multiple internal control genes. Genome Biol 3: RESEARCH0034 12184808 10.1186/gb-2002-3-7-research0034 PMC126239

[48] Andersen CL, Jensen JL, Orntoft TF. (2004) Normalization of real-time quantitative reverse transcription-PCR data: a model-based variance estimation approach to identify genes suited for normalization, applied to bladder and colon cancer data sets. Cancer Res 64: 5245–50. 15289330 10.1158/0008-5472.CAN-04-0496

[49] Kirschner MB, Edelman JJ, Kao SC, Vallely MP, van Zandwijk N, Reid G. (2013) The Impact of Hemolysis on Cell-Free microRNA Biomarkers. Front Genet 4: 94 10.3389/fgene.2013.00094 23745127 PMC3663194

[50] Guan Y, Chen L, Bao Y, Qiu B, Pang C, Cui R, et al (2015) High miR-196a and low miR-367 cooperatively correlate with unfavorable prognosis of high-grade glioma. Int J Clin Exp Pathol 8: 6576–88. 26261539 PMC4525873

[51] Cizeron-Clairac G, Lallemand F, Vacher S, Lidereau R, Bieche I, Callens C. (2015) MiR-190b, the highest up-regulated miRNA in ERalpha-positive compared to ERalpha-negative breast tumors, a new biomarker in breast cancers? BMC Cancer 15: 499 10.1186/s12885-015-1505-5 26141719 PMC4491222

[52] Zaharieva IT, Calissano M, Scoto M, Preston M, Cirak S, Feng L, et al (2013) Dystromirs as serum biomarkers for monitoring the disease severity in Duchenne muscular Dystrophy. PLoS One 8: e80263 10.1371/journal.pone.0080263 24282529 PMC3840009

[53] Kang K, Peng X, Luo J, Gou D. (2012) Identification of circulating miRNA biomarkers based on global quantitative real-time PCR profiling. Journal of animal science and biotechnology 3: 4 10.1186/2049-1891-3-4 22958414 PMC3415128

[54] Hermans MC, Hoeijmakers JG, Faber CG, Merkies IS. (2015) Reconstructing the Rasch-Built Myotonic Dystrophy Type 1 Activity and Participation Scale. PLoS One 10: e0139944 10.1371/journal.pone.0139944 26484877 PMC4618741

[55] Gonzalez-Barriga A, Kranzen J, Croes HJ, Bijl S, van den Broek WJ, van Kessel ID, et al (2015) Cell membrane integrity in myotonic dystrophy type 1: implications for therapy. PLoS One 10: e0121556 10.1371/journal.pone.0121556 25799359 PMC4370802

[56] Blondal T, Jensby Nielsen S, Baker A, Andreasen D, Mouritzen P, Wrang Teilum M, et al (2013) Assessing sample and miRNA profile quality in serum and plasma or other biofluids. Methods 59: S1–6. 10.1016/j.ymeth.2012.09.015 23036329

[57] Koutsoulidou A, Kyriakides TC, Papadimas GK, Christou Y, Kararizou E, Papanicolaou EZ, et al (2015) Elevated Muscle-Specific miRNAs in Serum of Myotonic Dystrophy Patients Relate to Muscle Disease Progress. PLoS One 10: e0125341 10.1371/journal.pone.0125341 25915631 PMC4411125

[58] McDonald JS, Milosevic D, Reddi HV, Grebe SK, Algeciras-Schimnich A. (2011) Analysis of circulating microRNA: preanalytical and analytical challenges. Clin Chem 57: 833–40. 10.1373/clinchem.2010.157198 21487102

[59] Koscianska E, Witkos TM, Kozlowska E, Wojciechowska M, Krzyzosiak WJ. (2015) Cooperation meets competition in microRNA-mediated DMPK transcript regulation. Nucleic Acids Res 43: 9500–18. 10.1093/nar/gkv849 26304544 PMC4627076

[60] Witwer KW. (2015) Circulating microRNA biomarker studies: pitfalls and potential solutions. Clin Chem 61: 56–63. 10.1373/clinchem.2014.221341 25391989

[61] Moldovan L, Batte KE, Trgovcich J, Wisler J, Marsh CB, Piper M. (2014) Methodological challenges in utilizing miRNAs as circulating biomarkers. J Cell Mol Med 18: 371–90. 10.1111/jcmm.12236 24533657 PMC3943687

[62] Perfetti A, Greco S, Bugiardini E, Cardani R, Gaia P, Gaetano C, et al (2014) Plasma microRNAs as biomarkers for myotonic dystrophy type 1. Neuromuscul Disord. 10.1016/j.nmd.2014.02.005 24679513

