# Supplementary figures and images for: Six Serum miRNAs Fail to Validate as Myotonic Dystrophy Type 1 Biomarkers
Source: PLoS One. 2016 Feb 26;11(2):e0150501. doi: 10.1371/journal.pone.0150501 (PMC4769077; doi:10.1371/journal.pone.0150501)

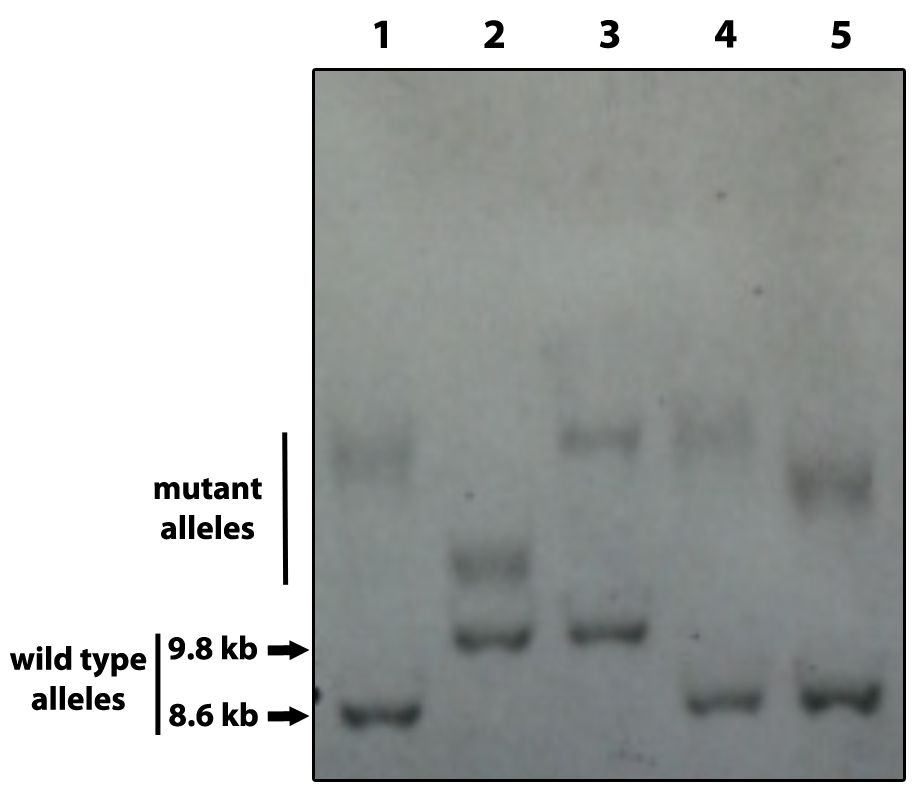

Supplement: S1 Fig — Arrows indicate wild type alleles. Mutant alleles in patients 1,3, 4 and 5 present more than 1000 CTG repeats while in patient 2 the mutant allele has a a repeat size of ~600 repeats. (TIF) [file pone.0150501.s001.tif]
